# Targeting tumor hypoxia and mitochondrial metabolism with anti-parasitic drugs to improve radiation response in high-grade gliomas

**DOI:** 10.1186/s13046-020-01724-6

**Published:** 2020-10-07

**Authors:** Faiqa Mudassar, Han Shen, Geraldine O’Neill, Eric Hau

**Affiliations:** 1 Translational Radiation Biology and Oncology Laboratory, Centre for Cancer Research, Westmead Institute for Medical Research, NSW Westmead, Australia; 2 Sydney Medical School, University of Sydney, NSW Sydney, Australia; 3 Children’s Cancer Research Unit, The Children’s Hospital at Westmead, NSW Westmead, Australia; 4 Children’s Hospital at Westmead Clinical School, Faculty of Medicine and Health, University of Sydney, NSW Sydney, Australia; 5 School of Medical Sciences, Faculty of Medicine and Health, University of Sydney, NSW Sydney, Australia; 6 Department of Radiation Oncology, Crown Princess Mary Cancer Centre, Westmead Hospital, NSW Westmead, Australia; 7 Blacktown Hematology and Cancer Centre, Blacktown Hospital, NSW Blacktown, Australia

**Keywords:** High-grade gliomas, Radioresistance, Hypoxia, HIFs, Mitochondria, Metabolism, Glioma stem cells, Anti-parasitic drugs

## Abstract

High-grade gliomas (HGGs), including glioblastoma and diffuse intrinsic pontine glioma, are amongst the most fatal brain tumors. These tumors are associated with a dismal prognosis with a median survival of less than 15 months. Radiotherapy has been the mainstay of treatment of HGGs for decades; however, pronounced radioresistance is the major obstacle towards the successful radiotherapy treatment. Herein, tumor hypoxia is identified as a significant contributor to the radioresistance of HGGs as oxygenation is critical for the effectiveness of radiotherapy. Hypoxia plays a fundamental role in the aggressive and resistant phenotype of all solid tumors, including HGGs, by upregulating hypoxia-inducible factors (HIFs) which stimulate vital enzymes responsible for cancer survival under hypoxic stress. Since current attempts to target tumor hypoxia focus on reducing oxygen demand of tumor cells by decreasing oxygen consumption rate (OCR), an attractive strategy to achieve this is by inhibiting mitochondrial oxidative phosphorylation, as it could decrease OCR, and increase oxygenation, and could therefore improve the radiation response in HGGs. This approach would also help in eradicating the radioresistant glioma stem cells (GSCs) as these predominantly rely on mitochondrial metabolism for survival. Here, we highlight the potential for repurposing anti-parasitic drugs to abolish tumor hypoxia and induce apoptosis of GSCs. Current literature provides compelling evidence that these drugs (atovaquone, ivermectin, proguanil, mefloquine, and quinacrine) could be effective against cancers by mechanisms including inhibition of mitochondrial metabolism and tumor hypoxia and inducing DNA damage. Therefore, combining these drugs with radiotherapy could potentially enhance the radiosensitivity of HGGs. The reported efficacy of these agents against glioblastomas and their ability to penetrate the blood-brain barrier provides further support towards promising results and clinical translation of these agents for HGGs treatment.

## Background

High-grade gliomas (HGGs) are malignant and incurable tumors arising from glial cells, present in the central nervous system (CNS). The World Health Organization (WHO) classifies the HGGs as either grade III or grade IV tumors, based on the histopathological criteria. HGGs can originate in various parts of the CNS and affect individuals of all age groups. Despite advances in treatment modalities, the heterogeneous nature of the tumor accounts for a notoriously poor prognosis and limits the efficacy of conventional therapies [1]. Resistance to chemo- and radiotherapy results in high relapse rates and tumor recurrence, thus posing a significant challenge to the field of oncology. The deadliest of these HGGs is the glioblastoma in adults and diffuse intrinsic pontine glioma (DIPG) in children.

### Glioblastoma

Glioblastoma is the most frequently diagnosed and aggressive tumor of all HGGs. It represents 14.6% of all primary CNS tumors and 48.3% of all primary malignant CNS tumors [2]. It is characterized as an anaplastic, poorly differentiated, and highly cellular, grade IV astrocytoma with the peak incidence between 45 and 70 years [3, 4]. The majority of HGGs (~ 95%) originate in the supratentorial area of the brain and a small proportion arise in the brainstem, spinal cord and cerebellum [5]. Despite optimal treatment, the median survival of glioblastomas is only 9–15 months, and the 5-year survival is 6.8%; thus, contributing to the significant social and medical health burden [2, 6].

Surgery is the primary standard of care for glioblastoma patients [7]. However, the infiltrative nature of glioblastoma means that it cannot be cured by surgery alone and approximately 80% of the tumors relapse within 2–3 cm margin of the pre-surgical lesion [3, 8]. Surgical resection is generally followed by radiotherapy, along with concomitant or adjuvant temozolomide (TMZ), an oral alkylating agent [3, 6]. The combination of radiation plus TMZ offers a modest survival advantage of 2.5 months only (14.6 months for combination treatment vs. 12.1 months for radiotherapy alone) and improving clinical outcomes in glioblastoma remains a challenge [9]. Moreover, despite decades of clinical research and investigating novel chemotherapeutic agents, glioblastoma remains incurable due to the complex nature of the tumor and intratumoral heterogeneity [8]. Radiotherapy remains the cornerstone of treatment for most glioblastoma patients [10], particularly where TMZ chemotherapy efficacy is limited. However, the intrinsic or acquired radioresistance of glioblastoma contribute to local tumor recurrence, thus limiting the effectiveness of radiotherapy [3].

### Diffuse Intrinsic Pontine Glioma

DIPG is a deadly form of HGG primarily affecting children. It represents 10–20% of all childhood brain malignancies and accounts for the majority of brain cancer-related deaths in children [11]. DIPG originates in the pons of the children and has a peak incidence between 6 and 9 years [11]. It is a fatal tumor as patients survive on average for only 8–12 months, with a subsequent increase in the mortality rate of 70% at one year, 90% at two years and > 99% at five years following diagnosis [12, 13].

As a result of the sensitive brainstem location and invasiveness of the tumor, DIPGs are not amenable to surgical resection [14]. Currently, radiotherapy is the only efficacious treatment available for prolonging patient survival [11, 15]. However, the effect of radiotherapy in controlling tumor size is temporary, and survival is only prolonged for approximately three months [16]. Symptoms of disease progression inevitably reappear 3–8 months following completion of radiotherapy [16]. Various chemotherapeutic agents, including TMZ, Bevacizumab (VEGF inhibitor) and EGFR inhibitors, have been investigated either alone or combined with radiation to improve patient prognosis [14, 17, 18]. Unfortunately, all the aforementioned strategies have been unsuccessful in improving the overall survival of DIPG patients over radiotherapy alone. The lack of efficacy of chemotherapy drugs might be as a consequence of the intrinsic resistance of DIPG to these drugs, or lack of ability of the drugs to penetrate the intact blood-brain barrier (BBB) [15, 19]. Other factors affecting the ability to deliver drug at sufficient concentration in DIPG include drug bioavailability, i.e. the serum levels and protein/tissue binding, blood flow to the pons, and the drug metabolism [14, 15].

Since all HGGs, including glioblastoma and DIPG, recur following treatment, there is an increasing need to develop new treatments in developing a cure for these deadly malignancies. Radiotherapy has been the mainstay of treatment for all malignant gliomas and has been the only standard treatment for DIPG for decades [1, 12]. However, after the completion of radiotherapy, almost all HGGs relapse secondary to radioresistance. Therefore, improving the effectiveness of radiotherapy and overcoming radioresistance remain the most promising approach to improve the patient survival outcomes. Understanding the mechanisms of radioresistance in HGGs may provide insight into ways to enhance the radiosensitivity of tumor cells and increase the efficacy of radiotherapy.

This review aims to outline mechanisms responsible for radioresistance in HGGs, particularly in glioblastoma and DIPG, with an emphasis on the role of hypoxia and the associated metabolic pathways implicated in tumor recurrence, metastasis, and resistance to chemo- and radiotherapy. It will address how inhibiting mitochondrial metabolism is an attractive strategy to decrease tumor hypoxia and increase oxygenation and therefore radiosensitize the tumor cells. It further discusses the need for mitochondrial targeted therapies against the resistant glioma stem cells (GSCs) which are significant contributors to tumor recurrence and relapse. Finally, it poses repurposing anti-parasitic drugs in combination with radiotherapy as an approach to abolish tumor hypoxia and eradicate both GSCs and glioma differentiated cells, and therefore offer an approach to improve radiotherapy efficacy.

## Hypoxia and Radioresistance

Cancer cells have various mechanisms to escape cell death via radiotherapy. Some of these mechanisms include, the ability of cancer cells to repair the radiation-induced DNA damage; cell-cycle arrest; alterations in the expression of oncogenes and tumor suppressor genes; autophagy induction; variations in tumor microenvironment such as hypoxia, elevation in cytokine levels, and epithelial to mesenchymal transitions; generation of cancer stem cells (CSCs); and alterations in tumor metabolism [20]. Of these, the development of tumor hypoxia and the associated metabolic pathways is one of the most important contributors to clinical radioresistance [21]. Hypoxia, a state which deprives the tissue of adequate oxygen supply, is a common microenvironment feature of almost all solid tumors [22]. Hypoxia is a pathophysiological condition that generally arises as a consequence of the rapid proliferation of cancer cells as they outgrow their blood supply, therefore depleting the cells of nutrients and available oxygen [22]. Hypoxic tumors are found to be highly aggressive, resistant to chemo- and radiotherapy, and associated with poor patient prognosis [23]. The hypoxic microenvironment poses a significant barrier and impedes the clinical outcome of radiotherapy as hypoxic tumors require triple the normal radiation dose to achieve the desirable cell death effect as irradiating normoxic tumors [24]. This indicates that tumor hypoxia substantially diminishes the efficacy of conventional anti-cancer approaches.

The link between hypoxia and radiation resistance was first established in the 1950s by Gray and his colleagues, who demonstrated that hypoxia caused radiation resistance in various plant, microbes and malignant mammalian tissues [25]. Ionizing radiation induces a chemical change and damages DNA by inducing free radicals [26]. Oxygen reacts with these free radicals, forming oxygen peroxide which “fix” the radiation-induced DNA damage, thus inducing permanent DNA damage [26]. As a consequence of the free radicals generated by radiation, an enormous amount of cytotoxic reactive oxygen species (ROS) are generated that interact with the DNA and cause further damage, leading to cell death [26]. However, under hypoxic conditions, DNA free radicals can restore back to their original form due to low oxygen levels, and irreversible DNA damage does not occur, thus compromising the radiation-induced DNA damage in the hypoxic tumor cells.

### Evidence of Hypoxia in HGGs

Hypoxia is associated with malignant progression, therapy resistance, and poor prognosis of glioblastomas [27, 28]. Previous research has provided experimental evidence of the presence of hypoxia in human gliomas [29, 30]. By utilizing an Eppendorf needle electrode, studies have revealed that oxygenation in glioblastoma drops to 10 mmHg compared to 40 mmHg in normal brain tissue [29, 30]. This provided solid support towards the underlying hypoxic radioresistance in gliomas which generally arises when oxygen levels in tumors drop to ~ 0–10 mmHg [30]. The close relationship between between hypoxia and radioresistance in gliomas has been noted in a number of studies [31–34].

A wide range of imaging approaches have been developed to understand the dynamics between oxygenation and hypoxia in gliomas, with positron emission tomography (PET) using ^18^F-fluoromisonidazole (FMISO) being the current gold standard for hypoxia imaging [27]. Using FMISO PET, Spence et al. 2008 showed that the volume and intensity of hypoxia in glioblastoma was associated with a poor time to tumor progression and reduced survival [35]. This identification of hypoxic regions in tumors could help in the design of modified dosages of radiotherapy or chemotherapeutics and therefore improve clinical outcomes [36].

Similarly, studies have also provided evidence of the possible link between hypoxia and pediatric HGGs and DIPG [37, 38]. Recently, Yeom et al. 2015 used proton magnetic resonance spectroscopy and identified high levels of citrate in DIPG patients which was correlated with poor tissue perfusion [38]. An increase in citrate, a metabolic by-product of the tricarboxylic acid (TCA) cycle has been previously observed in animal studies during hypoxic conditions [39]. Since tumor hypoxia relates to a decrease in blood supply, the hypoperfusion observed indicates that DIPGs are hypoxic tumors [38]. This hypoxic nature is the critical factor in conferring radioresistance to DIPGs [38]. Therefore, understanding the hypoxic mechanisms in cancers is crucial in order to derive strategies to target tumor hypoxia and improve the radiation response in HGGs.

## Role of Hypoxia and Mitochondrial Metabolism in Cancer Progression

### Hypoxia and HIFs

During hypoxic conditions, cancer cells upregulate crucial metabolic enzymes that help them adapt to the demand for nutrients and changes in their redox status. Adaptation to the hypoxic environment is partly mediated by the activation and stabilization of transcription factors termed as a hypoxia-inducible factors (HIFs) via inactivation of HIF propyl hydroxylases (PHD) [40]. HIFs comprise three isoforms with oxygen-labile α subunits (HIF-1α, HIF-2α, HIF-3α) that are involved in the adaptation to hypoxic stress, and a stable β subunit. Of these, HIF-1α is found to be constitutively expressed in many tumor cells, whereas the expression of HIF-2α and HIF-3α is restricted to certain tissues only. Under normoxia, HIF-α is stabilized via hydroxylation by PHDs, and undergoes ubiquitylation by binding to the tumor suppressor von Hippel-Lindau protein (pVHL), resulting in proteasomal degradation of the α subunit [40]. However, these HIFs are no longer degraded under hypoxia and instead, hypoxia causes HIFs to dimerize and bind to the hypoxia-responsive elements, thereby driving transcription of various genes vital for cancer cell survival, metabolism, angiogenesis, pH homeostasis, and metastasis [22]. Hypoxia dependent stabilization of HIFs is found to enhance tumor progression, metastatic dissemination, and maintain the intrinsically radioresistant GSC population [31, 32]. Hypoxia may sustain radioresistance of gliomas, and therefore targeting tumor hypoxia by inhibiting HIF-1α could enhance the radiosensitivity of human malignant gliomas and subsequently improve the radiotherapy outcomes [33, 34]. Moreover, hypoxia and HIFs overexpression in tumors is also correlated with increased risk of mortality [41].

### HIFs – Driver of Aerobic Glycolysis

**Fig. 1:**
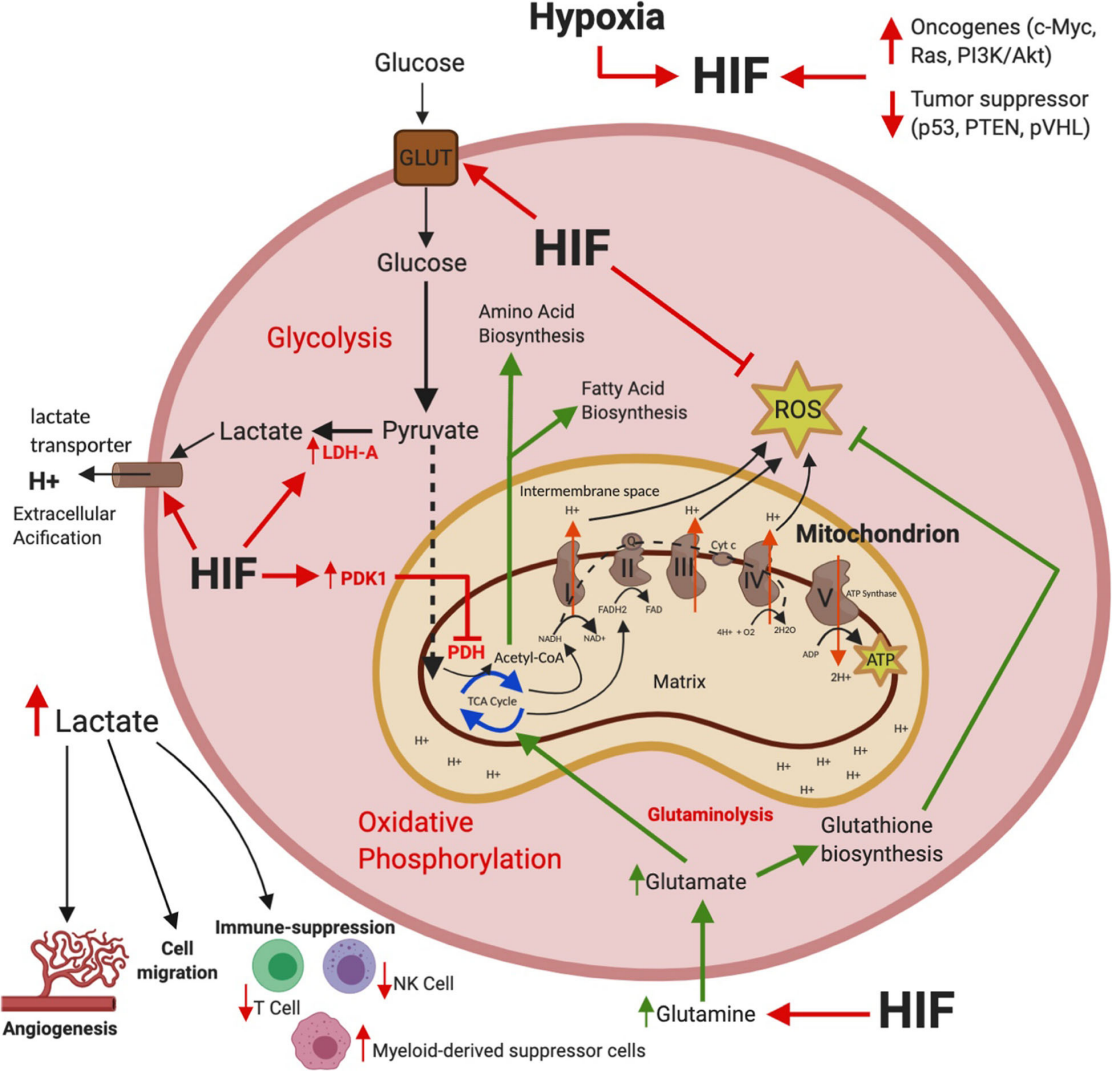
Schematic representation of the role of HIF in regulating glycolysis, glutaminolysis and OXPHOS. Variations in the status of oncogenes, tumor suppressor genes and hypoxia, are all important triggers of HIF-1α which drives cancer cells into glycolysis. Activation of HIF-1α increases glucose channeling into glycolysis by activating GLUT transporters and the key metabolic enzymes, lactate dehydrogenase A (LDH-A) and pyruvate dehydrogenase kinase 1 (PDK1). LDH-A drives pyruvate metabolism away from mitochondria, converting it into lactate. PDK1 inhibits mitochondrial pyruvate dehydrogenase (PDH) and therefore prevents pyruvate oxidation in mitochondria. HIF-1α also activates the lactate efflux transporter to remove excess lactate from the cytoplasm, resulting in an increase in extracellular acidification. By decreasing the pH of tumor microenvironment, lactate activates angiogenesis, cell migration and immune suppression pathways, therefore, providing a survival advantage to the tumor cells. Normal cells utilize pyruvate metabolism in mitochondria regulated by tricarboxylic acid (TCA) cycle. The products of TCA cycle, NADH, FADH2 provide electrons for the electron transport chain (ETC) chain. This process is known as oxidative phosphorylation (OXPHOS) and efficiently generates ATP. HIF-1α decreases mitochondrial OXPHOS by activating PDK1, which subsequently inhibits PDH. It also inhibits the excess reactive oxygen species (ROS) produced as a result of inefficient electron transport, therefore, protecting cancer cells against oxidative stress. Under hypoxic conditions and the consequent energy crisis, cancer cells also utilize glutamine to stimulate fatty acid and amino acid biosynthesis for energy production. HIF-2α enhances glutamine uptake which is converted into glutamate and replenishes the TCA cycle. The process of glutaminolysis generates fatty acids and amino acids as an energy source for cancer cells. Glutamate is also utilized for glutathione biosynthesis, which is a major antioxidant and quenches the ROS, therefore providing protection to cancer cells against cytotoxic ROS.

The metabolic requirements of normal healthy cells are considerably different from rapidly proliferating cancer cells. As opposed to normal cells that primarily depend upon the mitochondrial oxidative phosphorylation (OXPHOS) for energy production, most cancer cells preferentially use the aerobic glycolysis, converting glucose into lactic acid, even with enough available oxygen. Known as the Warburg effect, this results in abnormal proliferation of cancer cells and facilitates malignant progression [42]. The hypoxia induced transcription factors – HIFs, play a key role in driving cancer cells towards the glycolytic phenotype (Fig. 1) [42].

During aerobic glycolysis, the glucose converted into pyruvate is reduced to lactate within the cytoplasm, instead of undergoing OXPHOS in the mitochondria [22]. This response is mediated via HIF-1α driven gene expression of two metabolic enzymes - lactate dehydrogenase A (LDH-A) and pyruvate dehydrogenase kinase 1 (PDK1) [22]. LDH-A is vital in the conversion of pyruvate into lactate, and PDK1 inactivates pyruvate dehydrogenase (PDH) and subsequently prevents pyruvate oxidation in the mitochondria [22]. HIF-1α also controls lactate efflux via specialized monocarboxylate transporter – 4 (MCT4), as lactate production decreases cellular pH and is therefore toxic to cancer cells [43]. Hence, hypoxia and HIF-1α increase glucose channeling into glycolysis while suppressing OXPHOS and mitochondrial respiration by decreasing the input into mitochondria (Fig. 1) [44]. Gliomas are also found to preferentially rely on glycolysis and produce high concentrations of lactate [45]. The malignant phenotype of cancer cells is therefore supported by enhanced glycolytic rate, lactate production and excretion of lactate, resulting in extracellular acidification.

### Lactate – Signaling Molecule for Tumorigenesis

The production of large quantities of lactate as a by-product of glycolysis plays a pivotal role in regulating cancer phenotypes such as cancer cell migration, angiogenesis, metastasis, and immunosuppression [43]. Studies have demonstrated that lactate stimulates angiogenesis via HIF-1 mediated upregulation of the angiogenic growth factor and its receptor – VEGF and VEGFR2 by endothelial and tumor cells [46, 47]. Vegran et al. 2011 reported that lactate also modulates angiogenesis independent of HIFs by promoting NF-κB/IL-8 signaling [48]. Moreover, lactate also binds to N-MYC downstream regulatory gene 3 (NDRG3), which is highly induced under hypoxia and activates Raf-ERK pathway signaling, therefore promoting angiogenesis [49]. Extracellular lactate accumulation also promotes cancer cell migration by facilitating the interaction of CD44 and hyaluronic acid action on fibroblasts, therefore regulating cell adhesion, motility, and proliferation [50]. Interestingly, lactate also confers a survival advantage on cancer cells by enabling cells to escape immune surveillance [51]. This mainly occurs as lactate drives apoptosis of the cytotoxic T cells and natural killer (NK) cells and enhances the proliferation of myeloid-derived suppressor, cells which further decrease NK and T cell activity [51].

### Mitochondrial Biogenesis and Oxidative Phosphorylation in Cancers

An enhanced rate of aerobic glycolysis suggests that cancer cells should have a decreased rate of OXPHOS; however, current research increasingly suggests that along with glycolysis, some cancers also rely on mitochondrial biogenesis and OXPHOS for energy production and *in vivo* progression [52, 53]. The aforementioned is supported by the notion that cancer cells display high metabolic plasticity and can alter their metabolic phenotypes under various selection pressures [53]. A recent study by Shen et al. 2020 reported that although pediatric HGGs exhibit a glycolytic phenotype with reduced reliance on mitochondrial OXPHOS, inhibition of glycolysis by PDK1 inhibitor Dichloroacetate, stimulated OXPHOS by increasing PDH activity [54]. Another study found that various glioma cell lines had high dependency on mitochondrial OXPHOS for ATP production [55], and that glioma cells with preference for aerobic glycolysis could transition to OXPHOS under glucose deprived conditions [56]. This means that cancer cells can undergo metabolic reprogramming and are equipped with the ability to shift between glycolysis and OXPHOS depending upon the conditions in the tumor microenvironment.

The OXPHOS metabolic pathway involves the mitochondrial uptake of pyruvate being directed to the TCA cycle. This pathway regulates ATP production through the electron transport chain (ETC) complexes I-IV, present in the inner mitochondrial membrane [53]. The electron donors NADH, FADH_2_, and succinate, activate the ETC complexes, and the transport of electrons across the ETC complexes leads to the pumping of H + ions from the mitochondrial matrix to the intermembrane space [53]. Here, oxygen acts as the final electron acceptor and transports protons across the complex IV. This resulting proton gradient stimulates proton flow into the mitochondrial matrix through complex V - ATP synthase which generates ATP production (summarized in Fig. 1).

### Cancer Stem Cells and Mitochondrial Metabolism

CSCs, a subpopulation of cancer cells, contribute to tumor heterogeneity and play a vital role in tumor recurrence and metastatic dissemination [57]. These features of CSCs limit the efficacy of anti-tumor therapies and prevent uniform therapeutic effect across the entire tumor region due to the resistance to chemo- and radiotherapy [57–59]. CSCs undergo dynamic and reversible changes and exhibit a robust behavior due to functions including rapid DNA repair, resistance to oxidative stress, and adaptation to a hypo-nutrient microenvironment [60]. These resistant CSCs are highly dependent on mitochondrial biogenesis for survival and propagation [58, 61]. Vlashi et al. 2011 observed that unlike differentiated glioma cells, GSCs are less glycolytic and predominantly reliant on mitochondrial function and OXPHOS, producing large amounts of ATP [62]. These differences support the idea of inherent metabolic plasticity of cancer cells and the dynamic nature of cancer metabolism, which is highly influenced by factors such as the origin of cancer cells, mutational status, and nutrient availability in the tumor microenvironment [63]. This furthers our understanding of the radioresistance mechanisms of gliomas and suggests that there are two potential populations in HGGs that need to be targeted for complete eradication of the tumor i.e. GSCs and glioma differentiated cells. Of these, the resistant GSCs are more important as they could repopulate the tumor mass even after radiotherapy due to the inherent radioresistance. Since GSCs utilize OXPHOS and are generally spared by therapies targeted towards glycolysis, this suggests that using mitochondrial-targeted therapies may be a successful approach in the treatment of resistant and relapsed cancers by eliminating GSCs [62].

Importantly, subtypes of cancer cells within the heterogeneous tumor population could harbour CSC-like phenotypes due to metabolic symbiosis across neighboring cancer cells, promoting tumor development under unfavorable conditions [57]. The concept of metabolic symbiosis refers to the reuse of lactate produced from the hypoxic/glycolytic cells to fuel the ATP production in oxidative cells, thus lactate serves as an important contributor to OXPHOS [64]. Accumulating evidence suggests that both normal brain tissue and gliomas share metabolic symbiosis due to high expression of the two subtypes of monocarboxylate transporters - MCT4 and MCT1 which mediate the lactate shuttle across tumors [65]. Chemo- or radiotherapy induced redox stress could cause upregulation of HIF-1α which drives the activity of both MCT4- and MCT1- cancer cells [57]. The hypoxic tumor cells are MCT4-positive and undergo glycolysis. They exhibit a Warburg phenotype, producing excess lactate and secreting it via the MCT4. Lactate acts as substrate for oxygenated tumor cells which are MCT1-positive, displaying a ‘Reverse Warburg phenotype’ by converting the lactate back into pyruvate and providing fuel for mitochondrial OXPHOS. These MCT1-positive cancer cells have high mitochondrial abundance and behave similarly to cancer stem-like cells in the heterogeneous population of tumor [57, 66]. Since anti-tumor therapies function by increasing ROS, this allows MCT1-positive CSCs to continue to function via the mitochondrial pathway and maintain the tumor population, thus, preventing complete eradication of the tumor tissue [57]. Targeting MCT1 is reported to inhibit tumor growth and could render tumor cells sensitive to radiotherapy [66]. Since MCT1 is highly expressed in HGGs [67], targeting mitochondria and MCT1 could interrupt the symbiosis and decrease the ability of tumor to utilize the key metabolic substrates [64]. Additionally, inhibition of mitochondrial OXPHOS could increase tumor oxygenation and enhance the radiosensitivity of tumors, thus helping eradicate the robust CSCs population and improving treatment responses in cancers.

The tumor microenvironment plays a significant role in maintaining the stemness of CSCs. CSCs reside in niches with adjacent cells which provide growth factors, cytokines, and other extracellular matrix components, essential for tumor development and conserving the metabolic phenotypes of CSCs that underlie therapeutic resistance and distant metastasis [60]. A hypoxic microenvironment, with elevated expression of both HIF-1α and HIF-2α, is also reported to be advantageous for the survival and propagation of CSCs [60]. Studies show that GSCs reside amongst endothelial cells, forming a perivascular niche, where high osteopontin expression enables GSCs to acquire stem cell-like phenotypes and promotes radioresistance via the interaction of hypoxia-induced HIF-2α with the stem cell marker CD44 [68, 69]. This finding further establishes the link between tumor hypoxia and GSCs with radioresistance and suggests that targeting both of these elements is needed to improve radiation responses in gliomas.

## Targeting Tumor Hypoxia by inhibiting Mitochondrial Metabolism

Since tumor hypoxia is identified as a barrier to successful radiotherapy treatment, targeting hypoxia is an attractive approach to overcome radioresistance and enhance the effectiveness of radiotherapy [70]. In the past, research into alleviating tumor hypoxia was predicated on approaches that increase the oxygen supply to tumor regions, including oxygen delivery via hyperbaric oxygen chambers and using oxygen mimetics such as misonidazole and nimorazole which increase radiosensitivity via fixation of radical-induced DNA damage [71]. Other attempts involved the usage of hypoxia-activated prodrugs/compounds, including tirapazamine, N-oxides, transition metals, and quinones, that use enzymatic reduction reactions to form cytotoxic effector species which specifically eradicate hypoxic cells in tumors [72]. Despite their ability to decrease tumor hypoxia, these approaches did not yield significant clinical outcomes due to toxicity profiles, poor-perfusion and other limitations, and are therefore not used in clinical practice [71]. In contrast to the previous strategies, current research focuses on reducing oxygen demand by decreasing oxygen consumption rate (OCR) as an alternative approach to abolish tumor hypoxia [71]. Computational analysis also suggests that reducing OCR could be more efficacious in attenuating hypoxia and increasing oxygen concentration compared to previous methods of increasing oxygen delivery [73].

A decrease in OCR could be achievable by inhibiting the mitochondrial OXPHOS [53]. Mitochondria is a potential target for alleviating tumor hypoxia as mitochondrial inhibitors are found to decrease tumor cells’ demand for oxygen [74]. This occurs because oxygen is required for OXPHOS as it is the terminal electron acceptor in the mitochondrial ETC complexes (see Fig. 1), therefore, targeting ETC complexes could subsequently decrease the oxygen utilization and inhibit OXPHOS. As a result, there would be more oxygen available surrounding the tumor tissue and oxygen concentration would increase. As reoxygenation of radioresistant tumors would enhance their radiosensitivity and increase tumor cell death [70], targeting mitochondria would help abolish tumor hypoxia and therefore, improve the radiation response in HGGs. Moreover, this approach would also be effective in eradicating the radioresistant GSCs which do not respond to anti-glycolytic therapies and could further improve the efficacy of radiotherapy in these deadly malignancies.

## Other Roles of HIFs in Cancer Metabolism

### HIFs and Reactive Oxygen Species

Reduced oxygen tension also increases the production of ROS, therefore increasing cellular redox stress. Cancer cells counter this state by HIF1-mediated modulation of the subunits of ETC complex IV, also known as the cytochrome c oxidase (COX) present in the mitochondria [40]. Under normoxia, cells function by expression of the COX4-1 regulatory subunit [40]. However, hypoxia induces HIF-1 to upregulate the transcription of COX4-2 regulatory subunit and mitochondrial LON protease, which regulates the cleavage of COX4-1 subunit [44]. This subunit switch prevents inefficient electron transfer reactions and accumulation of toxic ROS, therefore, enabling cancer cells to survive under hypoxic microenvironment [40]. Hypoxia also slows down the ETC, therefore causing mitochondrial dysfunction by increasing the ratio of nicotinamide adenine dinucleotide and its oxidized state (NADH: NAD^+^) [40]. This is critical for the generation of mitochondrial redox couples – glutathione and thioredoxin, which quench the ROS, therefore, maintaining the stability and viability of cancer cells [40]. Low levels of ROS decrease the radiation-induced DNA damage and confers radioresistance to cancer cells.

### HIFs and c-MYC Upregulation

HIFs can also interact with proto-oncogenes such as c-MYC, altering tumor metabolism and driving malignant proliferation [44]. The interaction of HIF-1 and c-MYC directs cancer cells to glycolysis by upregulating the glucose transporter 1 (GLUT1) which increases glucose uptake, and hexokinase 2 (HK2) which catalyzes the first step of glycolysis, and PDK1 which suppresses mitochondrial respiration [75, 76]. Since hypoxia and HIF-1 promote the glycolytic phenotype, the relationship between HIF-1 and c-MYC shows that c-MYC allows cancer cells to adapt to hypoxic microenvironment [77]. Overexpression of c-MYC also maintains mitochondrial TCA cycle activity by activating HIF-2α mediated glutamine uptake as an alternative energy source and enhancing glutaminolysis, therefore, directing the available carbon into fatty acid, amino acid and nucleotide synthesis [44, 77]. Glutamine metabolism is repeatedly observed in various cancers as a compensatory pathway when cells are under an energy crisis [42]. Moreover, overexpression of MYC confers survival benefit to cancer cells by increasing the synthesis of biomolecules and energy production during hypoxic stress [44], thus signifying that it could be a potential drug target for anti-cancer therapies.

### HIFs and PI3K/Akt/mTOR Pathway

Besides hypoxia, HIF-1α is also regulated by the phosphatidylinositol – 4,5 – bisphosphate 3 – Kinase (PI3K)/protein kinase B (Akt)/mTOR pathway [78]. The PI3K/Akt pathway is involved in cell signaling, angiogenesis, cell proliferation, migration, and apoptosis [78]. It is upregulated in various cancers owing to several mutations such as loss of PTEN and plays a significant role in tumor development [78]. The mTOR pathway directly controls mitochondrial respiration and oxygen consumption, and mTOR inhibition result in a decrease in mitochondrial oxidative function [79]. The mTOR inhibition can also activate its downstream target AMPK, which is a sensor of the energetic status of cells and is known to control proliferation in some cancers [80, 81]. In addition to mTOR inhibition, inhibitors of PI3K/Akt can decrease HIF-1α expression, inhibit cell proliferation, and trigger cell death, and therefore are detrimental to tumor progression [78].

## Repurposing Drugs for HGG Treatment

Repurposing drugs for cancer treatment is a cost-effective approach as the knowledge of dosage, safety, and side effects profile is already available. An understanding of the radioresistance mechanisms of HGGs has revealed that both tumor hypoxia and GSCs are significant contributors to tumor recurrence and resistance to chemo- and radiotherapy. Since GSCs are highly dependent on mitochondrial biogenesis [62], using drugs that specifically target hypoxia and mitochondrial metabolism is an attractive strategy to abolish hypoxia and eradicate these resistant GSCs. Inhibition of these pathways would enhance the effectiveness of radiotherapy and could therefore ameliorate the survival outcomes of patients with HGGs.

Various anti-mitochondrial drugs that inhibit OXPHOS either directly or indirectly have been explored as agents for anti-cancer therapy. These include the mitochondrial complex I inhibitors – biguanides (metformin and phenformin), BAY 87-2243, IACS-010759, Fenofibrate, and VLX600; complex II inhibitors – Lonidamine, VLX600, and Alpha-tocopheryl succinate; complex III inhibitors – Atovaquone; complex IV inhibitors – Arsenic trioxide and VLX600; mitochondrial protein synthesis inhibitors – Tigecycline and Doxycycline; and mitochondrial transfer inhibitors – NOX2 inhibitors and CD38 inhibitors [82]. Studies into these anti-mitochondrial drugs found that biguanides such as metformin reduce OCR by 10–20%; however, the anti-parasitic drug, atovaquone, exhibited a more profound effect on OXPHOS inhibition as it reduced the OCR by greater than 80% [26, 83]. This leads to the assumption that anti-parasitic drugs with a similar mode of action may be more potent in abolishing tumor hypoxia and increasing radiosensitivity of tumor tissue and shifts the focus to understanding their underlying mechanisms.

**Fig. 2:**
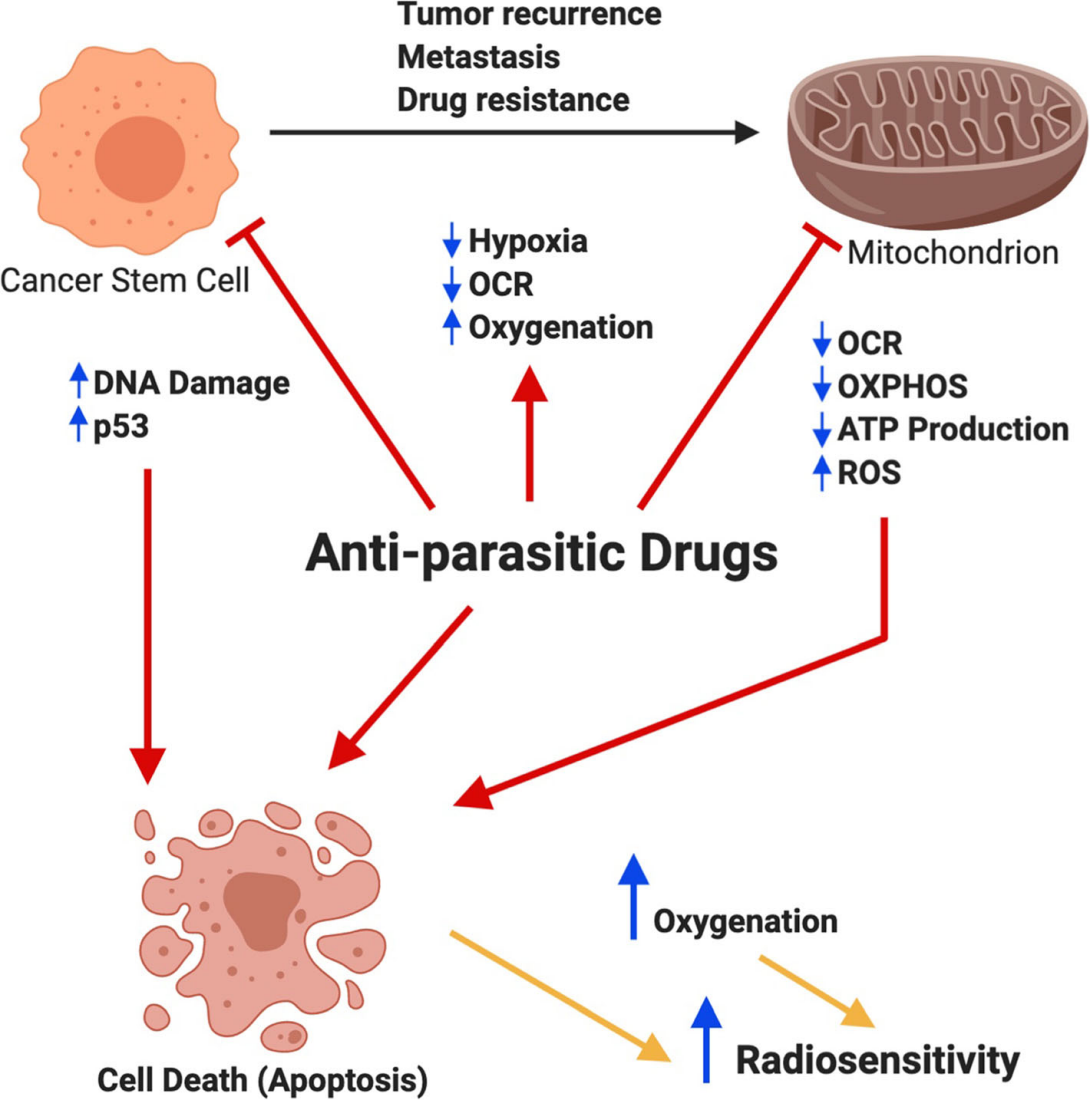
Targeting tumor hypoxia, mitochondrial function, and activating DNA damage pathways via repurposing anti-parasitic drugs. Cancer stem cells (CSCs) are highly dependent on mitochondrial function for propagation and are vital contributors to tumor recurrence, metastasis and resistance to chemo- and radiotherapy. Here, we propose the use of anti-parasitic drugs such as atovaquone, ivermectin, mefloquine, proguanil, and quinacrine to eradicate glioma stem cells (GSCs) and glioma differentiated cells by targeting various cancer metabolic pathways. These drugs alleviate tumor hypoxia and decrease the oxygen consumption rate (OCR) by targeting mitochondrial electron transport chain (ETC) complexes, subsequently inhibiting OXPHOS and enhancing oxidative stress. By decreasing hypoxia, these drugs could potentially increase oxygenation around the tumor tissue. Moreover, these drugs also upregulate mechanisms of DNA damage and tumor suppressor p53. As a consequence, these drugs are proposed to induce apoptosis of GSCs, which, along with an increase in oxygenation (due to reduction in tumor hypoxia), should enhance the radiosensitivity of tumor cells. Combining the anti-parasitic drugs with radiotherapy is therefore an attractive approach to increase oxygen availability and eradicate therapy resistant GSCs and enhance the efficacy of radiotherapy

Repurposing anti-parasitic drugs that have previously displayed promising results against various cancer types, including glioblastoma, may also yield substantial results against DIPG. Anti-parasitic drugs, such as atovaquone, ivermectin, mefloquine, proguanil, and quinacrine could inhibit tumor growth by targeting various cancer pathways. These drugs have the potential to alleviate tumor hypoxia by decreasing OCR, induce mitochondrial dysfunction and oxidative stress by inhibiting the mitochondrial ETC, and upregulate mechanisms of DNA damage; all of these would lead to an increase in cancer cell death. An increase in apoptosis of GSCs and reoxygenation of radioresistant HGGs by these drugs could increase the radiosensitivity of HGG tumor tissue and result in improved radiation response (summarized in Fig. 2). Apart from atovaquone, which has shown some promising radiosensitizing effect against hypopharyngeal carcinoma [84], the ability of other anti-parasitic drugs to sensitize tumor cells to radiotherapy have not been explored and this area is worth pursuing (see Table 1). A recent clinical trial identifies atovaquone as a hypoxia modifier in non-small-cell-lung carcinoma, suggesting that if atovaquone demonstrates a reduction in tumour hypoxia, it would pave a pathway to assess it in combination with radiation in further trials (trial number NCT02628080). Importantly, the inherent ability of these anti-parasitic drugs to penetrate the BBB further directs our attention to use them for research in the management of HGGs.

**Table 1:** Summary of the evidence of anti-neoplastic role of the anti-parasitic drugs: their current indications, cellular cytotoxic mechanisms of cancers, BBB penetration profile and radiosensitivity response

Drug Name	Indications	Mechanism of Action	Efficacy in Cancers	Efficacy in Brain Cancers	BBB Penetrability	Radiosensitivity	Source
Atovaquone	FDA-labelled: • Pneumocystis Pneumonia Non-FDA labelled: • Malaria • Toxoplasma Encephalitis • Toxoplasmosis • Babesiosis	• Mitochondrial dysfunction and oxidative stress • Akt/AMPK/mTOR pathway Inhibitor • STAT3 Inhibitor	• Breast Cancer • Hypopharyngeal carcinoma • Colorectal Carcinoma • Lung Carcinoma • Cervical Cancer • Retinoblastoma • Renal Cell Carcinoma • Acute Myeloid Leukemia • Thyroid Carcinoma	• Glioblastoma	Yes	• Hypopharyngeal Carcinoma	[84–92]
**Ivermectin**	FDA-labelled: • Infection by Onchocerca volvulus • Intestinal strongyloidiasis • Pediculosis Capitis • Rosacea Non-FDA labelled: • Ascariasis • Infection by Loa loa • Cutaneous larva migrans • Enterobiasis • Infection by Wuchereria bancrofti • Infestation by Phthirus pubis • Mansonelliasis • Scabies	• Mitochondrial dysfunction and oxidative stress • Akt/mTOR pathway inhibitor • Induces cytostatic autophagy by targeting PAK1/Akt axis • Induces chloride dependent membrane hyperpolarization • WNT/TCF pathway inhibitor • Induces DNA fragmentation and chromatin condensation • Inhibition of ROS-TFE3-dependent autophagy and enhancing apoptosis	• Renal Cell Carcinoma • Chronic Myeloid Leukemia • Breast Cancer • Ovarian Cancer • Colon Cancer • Leukemia • Melanoma • Oesophageal squamous cell carcinoma • Cervical Cancer	• Glioblastoma • Neuroglioma	Limited (increases with increase in concentration)		[93–104]
Mefloquine	FDA-labelled: • Malaria	• PI3K/Akt/mTOR inhibitor • Mitochondrial dysfunction and oxidative stress • Lysosomal disruption • Disrupts endolysosomal RAB5/7 • Targets B-catenin pathway • Inhibits NF-κB signaling • Induces autophagy and endoplasmic reticulum stress	• Prostate Cancer • Gastric Cancer • Cervical Cancer • Acute Myeloid Leukemia • Chronic Lymphocytic Leukemia • Colorectal cancer • Liver cancer • Breast Cancer	• Glioblastoma	Yes		[105–114]
Proguanil	FDA-labelled: • Malaria, Plasmodium Falciparum Non-FDA labelled: • Malaria, Plasmodium Vivax	• Mitochondrial dysfunction and oxidative stress	• Colon Cancer • Bladder Cancer		Yes		[115]
Quinacrine	Non-FDA labelled: • Malaria • Giardiasis • Tapeworm infection • Systemic Lupus erythematosus • Rheumatoid arthritis • Malignant pleural effusions • Prevention of recurrent pneumothorax • Female sterilization	• DNA intercalator, inhibits DNA repair pathways • FACT c-trapping • Induces apoptosis via TRAIL signaling, modulating topoisomerases, inhibiting NF-κB and inducing p53 • Arachidonic acid pathway inhibitor • Induces cytostatic autophagy and inhibits cytoprotective autophagy • Modulates cell cycle arrest	• Breast Cancer • Colon Carcinoma • Ovarian Cancer • Non-small cell lung cancer • Diffuse large B-cell lymphoma • Colorectal Cancer • Leukemia • Renal Cell Carcinoma • Melanoma • Gastric Cancer • Anaplastic Thyroid Cancer	• Glioblastoma	Yes		[116–127]

## Mechanism of Action of Anti-parasitic Drugs

### Atovaquone

Atovaquone, an anti-malarial drug, is FDA-approved for the treatment of lung infection called pneumocystis pneumonia, and for toxoplasmosis [85]. It is efficacious against a variety of protozoa such as *plasmodium* spp., *Toxoplasma gondii., P. carinii., and Babesia* spp. [128]. It was particularly developed to treat malarial infections and targeted the mitochondrial function in *Plasmodium* spp. by inhibiting the mitochondrial complex III, also known as cytochrome bc1 complex [85]. Atovaquone is ubiquinone (also known as coenzyme Q) analogue and blocks its binding site on cytochrome bc1 complex, therefore, inhibiting the transport of electrons into complex III [128]. This leads to a significant decline in mitochondrial membrane potential (MMP), disruption of key enzymes linked to the ETC, suppression of OXPHOS, and consequently results in a complete failure of malarial mitochondrial function [128]. Additionally, the anti-malarial activity of atovaquone is enhanced by synergizing it with proguanil [128].

Atovaquone is observed to decrease the OCR and alleviate hypoxia in various cancer cell lines by targeting mitochondrial complex III [84]. This effect has also been observed in animal models. Decreasing OCR is an attractive strategy to enhance the radiosensitivity of hypoxic tumors, and if reproduced in humans, it would render the tumors more sensitive to radiotherapy, thus improving clinical outcomes [26]. Fiorillo et al. 2016 illustrated that atovaquone reduces cell proliferation and induces apoptotic cell death in breast CSCs by inhibition of mitochondrial complex III and OXPHOS [85]. This resulted in a decrease in mitochondrial respiration, ATP levels, and MMP, along with a subsequent increase in cytotoxic ROS [85]. Moreover, atovaquone was found to decrease OCR and diminish hypoxia in FaDu (hypopharyngeal carcinoma), HCT116 (colorectal carcinoma) and H1299 (lung carcinoma) cell lines. It also reduced hypoxia in FaDu and HCT116 mouse xenografts models and significantly decreased tumor growth in the FaDu xenograft model in combination with radiation [84]. Other studies on the anti-cancer activity of atovaquone reveal that it induced apoptosis and inhibited cell growth in cervical cancer, thyroid cancer, retinoblastoma, and renal cell carcinoma (RCC), by suppressing mitochondrial respiration [86–89]. It also enhanced the sensitivity of retinoblastoma and RCC to chemotherapy and immunotherapy [88, 89]. Since mitochondrial biogenesis differs amongst cancers, the anti-cancer effect of atovaquone are more likely to eradicate those cancers with a higher dependency on mitochondrial biogenesis [86].

Atovaquone also targets signal transducer and activator of transcription 3 (STAT3), which is actively expressed in many solid and hematological malignancies [129]. Upregulated STAT3 contributes to tumorigenesis by increasing cancer proliferation, metastasis, drug resistance, and prevents apoptosis [129]. Atovaquone is found to inhibit STAT3 in thyroid cancer, acute myeloid leukemia (AML), and glioblastoma, therefore decreasing cell viability and inducing apoptosis in these cancers [87, 90, 91].

### Ivermectin

Ivermectin is FDA-approved for the management of onchocerciasis, intestinal strongyloidiasis, pediculosis capitis, and inflammatory lesion – rosacea [93]. It belongs to a family of macrocyclic lactone compounds called Avermectins, all of which bind to the glutamate-gated chloride ion channels (Glu-Cl) and interact with gamma-aminobutyric acid (GABA) gated Cl^−^ channels in the nerve and muscle cells of the parasites [130]. This results in an increased build-up of Cl^−^ ions, causing hyperpolarization of parasitic cell membranes, and leads to muscle paralysis and cell death [131]. The safety profile of ivermectin in mammals is confirmed by the presence of the intact BBB which secures GABA-sensitive neurons, therefore protecting against toxic effects of avermectins [131]. Ivermectin exhibits limited BBB penetrability due to the plasma membrane efflux pump p-glycoprotein which limits the drug intake into the brain [132]. However, a study found that avermectins, including ivermectin has the potential to inhibit p-glycoprotein efflux transporter and could penetrate the BBB at the appropriate concentrations [94, 133, 134]. This provides support for further research into the dosages required for BBB penetrability of ivermectin.

Interestingly, ivermectin has shown promising anti-cancer efficacy in various cancers by increasing mitochondrial dysfunction, inducing oxidative stress, and energy crisis [130]. By selectively targeting the ETC complex I, it inhibits the electron transport to the subsequent ETC complexes and causes a decline in MMP [130]. This results in a decrease in OCR, leading to suppression of mitochondrial respiration and ATP production, and also production of cytotoxic ROS which potentiate DNA damage [130]. A study by Zhu et al. 2017 observed that by targeting mitochondrial function, ivermectin causes apoptosis and suppresses cellular proliferation in a variety of RCC cell lines and also significantly impairs tumor growth in RCC xenograft mouse model [95]. Also, ivermectin-induced oxidative stress and mitochondrial dysfunction upregulated caspase-dependent apoptosis in chronic myeloid leukemia (CML) cells and inhibited tumor size in CML xenograft models [96]. It also sensitized the CML cells to BCR-ABL tyrosine kinase inhibitors, particularly, nilotinib and dasatinib [96]. Moreover, it exhibited preferential toxicity to both RCC and CML cells and spared the healthy cells due to the high reliance of these cancers on mitochondrial biogenesis [95, 96]. The inhibitory effects of ivermectin were also visualized in both *in vitro* and *in vivo* subcutaneous glioblastoma models, where it inhibited proliferation, induced caspase-dependent apoptosis, and potentiated angiogenic inhibition [97]. These responses were mediated via the role of ivermectin in depolarizing the MMP with an increase in ROS production, and inhibition of the capillary network formation [97].

Furthermore, ivermectin was also found to induce oxidative stress in glioblastoma cells by inhibiting the Akt/mTOR pathway [97]. The mTOR pathway is critical to mitochondrial function and acts as a cellular switch between glycolysis and mitochondrial respiration, such that mTOR inhibitors tend to decrease OCR and mitochondrial respiration directly and shift the cells towards a glycolytic phenotype [79]. The cytotoxic profile of ivermectin was not observed in mitochondrial-deficient cells or cells exposed to antioxidants, thus validating the role of ivermectin in inhibition of mitochondrial function [97]. Moreover, it induced caspase-mediated apoptosis and inhibited cell cycle progression in neuroglioma cells and suppressed tumor growth in *in vivo* xenografts [94].

Studies have also demonstrated that ivermectin induces cytostatic autophagy in breast cancer, and inhibits proliferation of ovarian cancer and oesophageal squamous cell carcinoma, by inhibition of PAK1 protein [98–100]. Moreover, it induces apoptosis in various other cancer such as melanoma by inhibition of ROS-TFE3 dependent autophagy; in leukemia by increasing chloride ions influx and ROS production; and in colon cancer cells via blocking the WNT/TCF pathway [101–103]. It also decreased cell viability of cervical cancer cells by mechanisms including mitochondrial dysfunction, increased ROS generation, DNA fragmentation, and chromatin condensation, and arresting cells in G1/S phase [104].

### Mefloquine

Mefloquine, a quinolinemethanol, is FDA-approved for the treatment of malarial infection caused by chloroquine-resistant *Plasmodium falciparum* and is an effective blood schizonticide for *P. vivax* [135]. It has been clinically available for thirty years and known to concentrate in the lysosomes of *P. falciparum;* however, the exact mechanism of action remains unknown. Recent investigation has found that mefloquine inhibits protein synthesis by interacting with the GTPase-associated center of the 80S ribosomal subunit in *P. falciparum* and therefore kills the malarial parasite [136].

Mefloquine has demonstrated anti-cancer efficacy across various cancer cell lines and has been repurposed in the therapeutic targeting of several cancer signaling pathways. Yan et al. 2013 observed that mefloquine reduced the cell viability of prostate cancer cells by altering MMP and producing excess cytotoxic ROS, which decreased the phosphorylation of Akt and triggered JNK, ERK and AMPK signaling pathways [105]. Interestingly, mefloquine did not inhibit the proliferation of normal human fibroblasts, thus indicating that it selectively targets the cancer cells [105]. Another study reported that mefloquine prevents the proliferation of the gastric cancer cells and reduced tumor growth in xenograft mouse models by inhibition of PI3K/Akt/mTOR pathway [106]. Similarly, it also displayed cytotoxicity against cervical cancer cells by targeting mitochondrial function and deactivating the mTOR pathway [107]. An increase in levels of PARP cleavage protein, which inhibits PI3K/Akt system, was also observed [107]. The PI3K/Akt pathway is critical for protecting MMP as it inactivates pro-apoptotic BAD protein and reduces cellular oxidative stress [137]. Activation of the Akt system also phosphorylates various downstream regulatory proteins such as BAD, caspase-9, FKHR, and GSK3β, which protects against cellular stress [137]. Similarly, the mTOR pathway is highly sensitive to mitochondrial dysfunction, and inhibition of this pathway results in a decrease in OCR, mitochondrial respiration, and overall mitochondrial activity [79]. Hence, targeting the PI3K/Akt/mTOR pathway in cancer cells would directly influence the mitochondrial function and trigger cell death.

Mefloquine also stimulated apoptotic cell death in breast cancer by inhibiting autophagy and decreased the proliferative and self-renewal capacity of liver CSCs by targeting the β-catenin pathway [108, 109]. It inhibited tumor proliferation and decreased tumor growth in colorectal cancer xenograft mouse models by targeting NF-κB and the downstream signaling pathways [110]. It further prevented autophagic degradation of defective mitochondria in colorectal CSCs by suppression of RAB5/7, LAMP1/2, and PINK1/PARKIN, therefore resulting in increased mitochondrial dysfunction and cellular apoptosis [111].

Furthermore, mefloquine was also found effective in inducing cell death in AML, chronic lymphocytic leukemia (CLL), and glioblastoma through disruption of lysosomes [112–114]. Lysosomal disruption releases the catalytic proteases cathepsins into the cytosol, which activates mechanisms of apoptosis [113]. Mefloquine stimulates the release of cathepsins from lysosomes in AML and initiates mitochondria-mediated cell death [113]. In glioblastoma, mefloquine disturbed the lysosomal stability and increased the levels of caspase-3, inducing cell death regardless of the p53 status [114]. It was also found to be more potent than chloroquine at inducing glioblastoma cell death and exhibited a better BBB penetrability profile [114].

### Proguanil

The biguanide proguanil was first developed as an anti-malarial drug for the management of acute *P. vivax* malaria; it was initially used alone and later administered in combination with chloroquine or atovaquone and the combination was found to be highly effective [138]. It is metabolized into cycloguanil, which regulates inhibition of parasite dihydrofolate reductase [138]. Another synergistic combination of proguanil and atovaquone is effective against *P. falciparum* malaria; both of these drugs target the parasite *P. falciparum’s* pre-erythrocytic and erythrocytic stages and induces causal and suppressive prophylaxis [139].

Proguanil enhances the activity of atovaquone in collapsing MMP and causing mitochondrial dysfunction [139]. Despite of being a complex I inhibitor; proguanil does not inhibit cellular and mitochondrial respiration as its penetrability into the mitochondria is limited [140]. However, proguanil still displayed the highest growth inhibition of colon and bladder cancer cell compared to other biguanides [115]. This points to the intriguing possibility of the extramitochondrial cytotoxic activity of proguanil and also suggests its potential role as an anti-cancer agent [115].

### Quinacrine

Quinacrine, an acridine derivative, was initially used for the prophylaxis and management of the malarial infection [141]. It is clinically available as quinacrine dihydrochloride and is effective against the treatment of various conditions, including malaria, giardiasis [142], and tapeworm infection [143]. Quinacrine’s effectiveness has also been reported in autoimmune inflammatory conditions, including systemic lupus erythematosus [144], and rheumatoid arthritis [145]. Moreover, it is efficacious in the treatment of pleural effusions in cancer patients [146] and also prevents spontaneous pneumothorax in patients with increased recurrence risk [147]. Quinacrine has also been utilized for safer female sterilization as it causes fibrosis and occlusion of fallopian tubes [148], and is currently under investigation for managing Creutzfeldt-Jacob disease [149].

The anti-cancer potential of quinacrine has been extensively explored, revealing various therapeutic targets. Due to its low toxicity profile, minimal side effects, and interference with many cancer signaling pathways, quinacrine offers compelling evidence as a promising antineoplastic agent [116]. The cytotoxic mechanisms of quinacrine include its ability to intercalate into DNA and insert between adjacent base pairs resulting in DNA damage [150]. It also prevents DNA damage repair by interfering with the nuclear proteins and inhibits both DNA and RNA polymerases, topoisomerases, telomerase, and upregulates tumor necrosis factor-related apoptosis-inducing ligand (TRAIL) therefore promoting apoptosis [116]. Quinacrine stimulates TRAIL binding with death receptors 4 and 5 (DR4 and DR5) and upregulates mitochondrial intrinsic apoptotic cascade [116]. It abrogates the arachidonic acid pathway by directly targeting the enzyme phospholipase A2 (PLA2), resulting in a decline in production of prostanoids (COX), leukotrienes (LOX), and eicosanoids (MOX/CYP450), as these prevent cell death and sustain cancer cell survival [150]. Moreover, quinacrine intercalates into DNA and alters the chromatin structure by inducing FACT (facilitates chromatin transcription) complex chromatin trapping; this leads to suppression of the NF-κB pathway and phosphorylation of the tumor suppressor protein p53 [116]. Hence, quinacrine has the potential to be effective against various cancers by targeting single or multiple signaling pathways and inducing apoptosis. The DNA damaging potential of quinacrine makes it an effective candidate to be combined with radiotherapy and could significantly enhance the efficacy of radiotherapy in HGGs.

A study by Preet et al. 2012 demonstrated that quinacrine targets breast cancer cells and induces cell death, cell cycle arrest, and decreases cell migration through inhibition of topoisomerases and increased DNA damage [117]. It stimulates apoptotic pathways in colon cancer cells by increasing the activity of p53 and p21 and the associated pathways [118]. Research has also found that quinacrine induces autophagic and apoptotic cell death and suppressed tumor growth in ovarian cancer by downregulating p62/SQSTM1 [119]. Furthermore, quinacrine was found to be effective in enhancing apoptosis in diffuse large B-cell lymphoma by downregulating MSI2 NUMB signaling pathway, suppressing c-Myc and arresting cells in S phase [120]. It exhibited a significant tumoricidal effect against RCC by inhibiting NF-κB and activating p53; reduced cell viability and decreased tumor volume in melanoma xenograft mouse models; induced apoptosis in leukemia cells by depolarization of mitochondria, oxidative stress and downregulation of Bcl-2 and Bcl-2L; and inhibited cell growth and cell cycle progression in gastric cancer cells via activating p53 and caspase-3 dependent apoptotic pathways [121–124].

Quinacrine has also been found to act synergistically with chemotherapy drugs such as carmustine, oxaliplatin, and carboplatin, or inhibitors including cisplatin, 5-fluorouracil, paclitaxel, and sorafenib, across various cancer types [119, 125, 151–155]. It is useful in overcoming resistance against tyrosine kinase inhibitor erlotinib in non-small cell lung cancer via inhibition of NF-κB, FACT, and induction of cell cycle arrest [126]. Moreover, quinacrine inhibited autophagy and reduced the cell viability of human glioblastoma cells both alone and in combination with the kinase inhibitor SI113 by upregulating p62 [127]. Another study by Wang et al. 2017 discovered that quinacrine induced apoptosis in GSCs and enhanced the efficacy of curcumin in eradicating glioma cells both *in vitro* and *in vivo* animal models [156]. Due to its ability to penetrate the BBB, induce DNA-damage and apoptosis in cancers, and its efficacy in gliomas, quinacrine seems a promising therapeutic agent for treatment of glioblastoma and DIPG. The DNA damaging role of quinacrine indicates that combining it with radiotherapy could be an effective approach in prolonging patient survival.

Quinacrine was later replaced by chloroquine for the treatment of malaria, however, it continued to be used in the treatment of diseases. Chloroquine, as an anti-malarial agent, has also shown promising anti-cancer potential. It is found to disrupt autophagy by blocking the formation of autolysosomes and activating the GRP78/BiP chaperone which enhances endoplasmic reticulum (ER) stress [157, 158]. Impairment of autophagy results in apoptosis and this could therefore sensitize cancer cells to chemo- and radiotherapy [157]. Interestingly, chloroquine has been shown to act synergistically with TMZ by inducing ER stress [158].

## Conclusion and Future Directions

Glioblastoma and DIPG are deadly and malignant forms of HGGs, contributing to the medical health burden. Despite decades of research and investigating novel agents in clinical trials, both tumors result in high mortality rates and no cure has been established. Patients undergoing radiotherapy experience high rates of recurrence due to the intrinsic radioresistance of the glioma cells. Therapies targeting radioresistance mechanisms are urgently needed to improve radiation response of HGGs as these could increase the overall survival of patients. Tumor hypoxia and alterations in the metabolic status enable cancer cells to achieve the malignant and resistant phenotype. Targeting hypoxia and increasing oxygenation around the tumor tissue is essential for the effectiveness of radiotherapy in HGGs. Inhibiting mitochondrial metabolism could help abolish tumor hypoxia by decreasing the OCR of tumor cells and could subsequently increase the oxygen availability. Therapies targeted towards mitochondria would also be beneficial against GSCs which preferentially rely on mitochondrial metabolism and are key players in tumor recurrence and resistance to therapies [62]. Therefore, developing strategies that specifically target tumor hypoxia and inhibit mitochondrial function, or combination strategies to target both glycolysis and OXPHOS, may be beneficial increasing oxygenation and inducing apoptosis of both GSCs and differentiated glioma cells and could improve the efficacy of conventional and biological therapies [54].

There is considerable evidence supporting the anti-neoplastic role of anti-parasitic drugs (summarized in Table 1). Increasing evidence of the role of anti-parasitic drugs in inducing mitochondrial dysfunction and activating DNA damage pathways, makes them attractive candidates for increasing the radiosensitivity of HGGs. Since these drugs have yielded efficacious results against glioblastoma either alone or in combination with other compounds, this points to the intriguing possibility of their effective role in both glioblastoma and DIPG. Further research should consider investigating these drugs for the treatment of HGGs in combination with radiotherapy. The rationale behind increasing radiosensitivity of glioma cells is via reoxygenation of hypoxic tumor tissue and inducing apoptosis of GSCs, therefore, improving efficacy of radiotherapy (Fig. 2).

A significant limitation in the current literature is that these anti-parasitic drugs have only been investigated in *in vitro* cancer cell lines and patient-derived xenograft (PDX) implanted in immunocompromised mice. It is important to consider that the successful development of anticancer therapies must consider the interaction between the tumor cells and immune cells. Since the immune system poses a major challenge to the long-term success of the anti-cancer therapies, one possible avenue could be to investigate the antineoplastic role of these drugs in ‘humanized’ mouse models that closely mimic the patient tumor microenvironment [159]. Therefore, future research should also consider exploring this area for both glioblastoma and DIPG therapy.

In summary, the combination of anti-parasitic drugs and radiotherapy might serve as novel approach towards the management of malignant HGGs and may improve patient survival outcomes. There is need to study the effects of these compounds on interactions between cancer cells and immune system as this could provide further insight into the translation of this approach to clinical practice.

## Data Availability

Not applicable.

## Abbreviations

HGG: High-grade glioma
CNS: Central nervous system
DIPG: Diffuse intrinsic pontine glioma
TMZ: Temozolomide
BBB: Blood brain barrier
GSC: Glioma stem cell
CSC: Cancer stem cell
ROS: Reactive oxygen species
PET: Positron emission tomography
TCA: Tricarboxylic acid cycle
HIF: Hypoxia-inducible factor
PHD: Prolyl hydroxylase
OXPHOS: Oxidative phosphorylation
LDH-A: Lactate dehydrogenase A
PDK1: Pyruvate dehydrogenase kinase 1
PDH: Pyruvate dehydrogenase
MCT: Monocarboxylate transporter
ETC: Electron transport chain
OCR: Oxygen consumption rate
COX: Cytochrome c oxidase
MMP: Mitochondrial membrane potential
RCC: Renal cell carcinoma
STAT3: Signal transducer and activator of transcription 3
CML: Chronic myeloid leukemia
AML: Acute myeloid leukemia
CLL: Chronic lymphocytic leukemia
