# Correction to: Targeting tumor hypoxia and mitochondrial metabolism with anti-parasitic drugs to improve radiation response in high-grade gliomas

**DOI:** 10.1186/s13046-021-02161-9

**Published:** 2021-12-01

**Authors:** Faiqa Mudassar, Han Shen, Geraldine O’Neill, Eric Hau

**Affiliations:** 1 Translational Radiation Biology and Oncology Laboratory, Centre for Cancer Research, Westmead Institute for Medical Research, Westmead, NSW Australia; 2 Sydney Medical School, University of Sydney, Sydney, NSW Australia; 3 Children’s Cancer Research Unit, The Children’s Hospital at Westmead, Westmead, NSW Australia; 4 Children’s Hospital at Westmead Clinical School, Faculty of Medicine and Health, University of Sydney, Sydney, NSW Australia; 5 School of Medical Sciences, Faculty of Medicine and Health, University of Sydney, Sydney, NSW Australia; 6 Department of Radiation Oncology, Crown Princess Mary Cancer Centre, Westmead Hospital, Westmead, NSW Australia; 7 Blacktown Hematology and Cancer Centre, Blacktown Hospital, Blacktown, NSW Australia

**Correction to: J Exp Clin Cancer Res 39, 208 (2020)**

**https://doi.org/10.1186/s13046-020-01724-6**

Following publication of the original article [1], Han Shen is also affiliated to Affiliation 1: Translational Radiation Biology and Oncology Laboratory, Centre for Cancer Research, Westmead Institute for Medical Research, NSW, Westmead, Australia.

The correction does not have any effect on the results or conclusions of the paper. The original article has been corrected.
